# Influence of dietary protein levels on nano-encapsulated *Yucca schidigera* extract and its effects on in vitro ruminal greenhouse gas production and fermentation dynamics

**DOI:** 10.1038/s41598-025-94238-0

**Published:** 2025-03-18

**Authors:** Edwin Oswaldo Botia-Carreño, Mona M. M. Y. Elghandour, Akaninyene Jack, Udoh A. Inyang, Susanne Kreuzer-Redmer, Abdelfattah Z. M. Salem

**Affiliations:** 1 Facultad de Medicina Veterinaria y Zootecnia, Universidad Nacional Autónoma de México, Avenida Universidad 3000, 04510 Ciudad de México, Mexico; 2 Facultad de Medicina Veterinaria y Zootecnia, Universidad Autónoma del Estado de México, El Cerrillo Piedras Blancas, 50295 Toluca, Estado de México Mexico; 3 Department of Animal Science, Faculty of Agriculture, University of Uyo, Uyo, Akwa Ibom State Nigeria; 4 Centre for Animal Nutrition and Animal Welfare Sciences, Clinical Department for Farm Animals and Food System Safety, University of Veterinary Medicine Vienna, Veterinärplatz 1, 1210 Vienna, Austria; 5 Dipartimento di Scienze del Suolo, della Pianta e degli Alimenti (Di.S.S.P.A.), Università degli Studi di Bari, Via Giovanni Amendola, 165/A, 70126 Bari, BA Italy

**Keywords:** Nanoencapsulation, *Yucca schidigera* extract, Greenhouse gases, Ruminants, Protein-varied diets, In vitro, Biological techniques, Biotechnology, Environmental sciences, Nanoscience and technology

## Abstract

The influence of nano-encapsulated *Yucca schidigera* extract (YSE) on total gas (GP), ruminal methane (CH_4_), carbon monoxide (CO), hydrogen sulphide (H_2_S) production, and fermentation activities of diets based on two different protein levels were investigated. A completely randomized experimental design with a factorial arrangement (2 × 4 × 4) with three replications was used. Factor 1 was the dietary protein levels (14%and 18%), factor 2 was the types of extracts used (TE; negative control (without extract), positive control (empty chitosan nano-capsules), *Y. schidigera* extract nano-capsules, and crude *Y. schidigera* extract), and factor 3 the doses of each type of extract (ED; 0-, 0.25-, 0.5-, and 1.0- mL extract/g DM). Nano-chitosan reduced the GP production with a higher protein level by 24.9% after 48 h while the crude extract elevated it. At both crude protein levels, the interaction of crude extract at an ED of 0.25 mL extract/g DM generated a higher volume of CH_4_ at 6 h (*p* = 0.001 and 0.001 respectively) compared to the volume generated by the negative control. The 0.25 mL extract/g DM extract for both the crude extract and nano-extract elicited higher and lower CO production at 6 h (*p* < 0.0001), respectively. Nano-chitosan at 0.25 mL extract/g DM resulted in less H_2_S produced at 6 h than when crude extract was used at the same dose and a higher protein level (*p* = 0.027). The ED did not significantly affect any of the parameters under consideration as used under lower protein levels. However, TE affected pH and dry matter degradability (*p* < 0.0001) while the interaction of both TE and ED impacted both CH_4_:SCFA and CH_4_:ME (*p* = 0.045) with higher and lower values obtained for nano-chitosan and the negative control, respectively. In conclusion, nano-chitosan at a higher protein level proved its antimicrobial property, and although the production of CO increased at 14% protein, in vitro fermentation indicated its ability to minimize the production of GP, methane and hydrogen sulphide in the rumen, and to boost the degradability of DM and methane conversion efficiency.

## Introduction

Inefficiency in feed resource utilization by ruminants is one of the frontline concerns in ruminant production. Firstly, it constitutes an energy loss to the animal via methane (CH_4_) production and emission thereby impacting negatively on production. About 15% of energy loss by ruminants from ingested feed resources in the form of CH_4_ translates into an enormous amount of energy wastage considering the quantity emitted *per* ruminant, and the fact that a kilogram of CH_4_ contains 50–55.5 MJ of energy^1^. Secondly, its contribution to environmental degradation is substantial; Methane and carbon dioxide (CO_2_) are contributors to global warming, and these are the major gases produced from feed fermentation in the rumen with carbon monoxide (CO) and hydrogen sulphide (H_2_S) being of minor production. Methane and H_2_S are also alternative sinks for H_2_ generated from the degradation of fibrous feed by ruminants. These inefficiencies can be attributed to but are not limited to several interconnected factors such as digestive system complexity^2^, feed quality^3^, feed formulation^4^, health issues^5^, and environmental aspects^6^. Severally attempts including for instance the use of enhanced feed formulation, the inclusion of precision nutrition with additives to improve nutrient digestion, degradation, and absorption^7^, genetic selection^8^ and technological innovations^9^ have been advanced to address the challenge of feed resource utilization, to promote more sustainable and profitable ruminant production systems. However, strategic approaches that are goal- and cost effective, sustainable and also simple to use will go a long way. One area where ruminant nutritionists have concentrated on has been the use of feed additives to improve efficiency of feed resource utilization leading to better animal performance, reducing feed costs, and lower environmental impacts. Feed additives such as enzymes^7^, probiotics and prebiotics^10,11^, ionophores^12^, buffers and acidifiers^13^, plant extracts, and essential oils^14^ have contributed to enhancing feed efficiency. Feed additives are primarily substances with antimicrobial activity^15^ and noting that the (prophylactic) use of antibiotics has been of global concern due to the formation of antimicrobial resistance^16^, where alternative agents are urgently needed. This study emphasizes the usage of two natural compounds that possess antimicrobial properties and could be utilized to manipulate the rumen microbial ecosystem and in turn improve feed resource utilization.

*Yucca schidigera* is a desert plant native to the arid areas of Mexico^17^ and its major active components used as animal feed additives are the steroidal saponins. The positive impacts of yucca crude extract in defaunation on rumen metabolism have made it of growing interest. Moreover, the increase in the generation of volatile fatty acid by *Y. schidigera* accompanied by decreased methane generation^18^, is an indication of its potential to alter the population of the microbial communities with a resultant change in the carbon balance of the rumen for better digestion of carbohydrates^19^. Its usage has shown some level of success, but contrary results as reported by Zijderveld et al.^20^ and Li^21^ have been attributed to diet/substrate^22^, incubation time^23^, and composition of active ingredients^24^.

Chitosan is commonly obtained from chitin, which is found in the shells of marine crustaceans and cell walls of fungi. Chitosan has been used as a hemostatic agent, antioxidant, antimicrobial agent, anti-cholesterolemic agent, drug delivery carrier, biosensor, antitumor agent, for immunogenicity, and as anti-coagulant^25^. Chitosan’s wide acceptance has made it a useful component for numerous agricultural and biological applications^26^. It is edible and demonstrates significant nutritional potential when incorporated as a feed additive^27^. Thus, feed formulations with the inclusion of chitosan and its related composites have been of growing interest in the nutrition of animals. Researches have elicited a positive response in immunology, productivity, growth performance, and also inhibition of microbial pathogens in the intestine^28^ as well as methanogenesis^29^. Chitosan-supplemented diets could be an alternative to antibiotics, alongside significant improvements to gut functions^30^. Several researchers have stated the drug-delivering properties of chitosan, where samples were used as carriers for the efficient loading, targeted delivery, and controlled release of active substances to the target sites^31,32^. However, the mechanisms of action of chitosan and its impacts on the microbial habitat of the rumen still need to be evaluated for the optimal benefits that could be derived from its use as a feed additive.

Numerous benefits can be derived from the use of bioactive ingredients such as polyphenols, essential oils, antioxidant agents, vitamins, and microbial feed additives except that their use is limited by their unpleasant taste, relatively low solubility, chemical instability, rapid release time, poor bioavailability, or susceptibility to oxidative degradation^33,34^. This necessitates the use of these biological composites in forms such as nano capsules, nanogels, and microbeads, other than their crude form, for their protection and optimal benefit. Previous in vitro studies primarily focused on energy-based feedstuffs and/or silages, with limited investigation into the effects of varying CP levels. Diets formulated with different protein levels could reveal diverse effects on greenhouse gas emissions, providing valuable insights into how protein content influences the production of gases such as methane, carbon dioxide, and hydrogen sulphide^35^. For instance, a study by Schrade et al.^36^ demonstrated that reducing dietary CP levels significantly decreased ammonia and nitrous oxide emissions in dairy cattle, highlighting the impact of protein content on greenhouse gas emissions.

A combination of the two additives has not yet been studied and it was in view of this, that the influence of nanoencapsulated *Yucca schidigera* extract on ruminal methane, carbon monoxide and hydrogen sulfide production and fermentation activities of two protein levels-based diet was investigated in this work.

## Materials and methods

### Nanoencapsulation of *Y. schidigera* extract

The nonencapsulated *Yucca schidigera* extract (i.e., YSE) was made using Chitosan as the encapsulating biopolymer (supplier: Sigma-Aldrich, Toluca, Mexico). A *Yucca schidigera* extract from the Bioliquid 3000 brand with a concentration of 97.9%, manufactured in Baja California, northwest of Mexico by the company Baja Agro International S.A. de C.V. AGROIN, was used. *Yucca* *schidigera* and its saponin-rich extracts are GRAS certified by the FDA for human use^37^. *Yucca schidigera (Agavaceae*) is one of the main commercial sources of steroidal saponins; additionally, the presence of resveratrol and some new phenolic compounds with a very unusual spiral structure, named yuccaols A–E and yuccaone A, has been found by Piacente et al.^38^; The encapsulation of the *Yucca schidigera* extract was carried out in two phases using 100 mL of a 1% acetic acid solution. In phase one, 0.5 g of Pluronic F127 (Sigma-Aldrich, Toluca, Mexico) was weighed and gradually added to 50 mL of the 1% acetic acid solution under constant agitation until completely dissolved, and then 0.3 g of chitosan (Sigma-Aldrich, Toluca, Mexico) was added.

In phase two, 0.1 g of sodium tripolyphosphate (Sigma-Aldrich, Toluca, Mexico) was weighed and added to the remaining 50 mL of the 1% acetic acid solution until completely dissolved, and then 0.18 mg of *Yucca schidigera* extract was gradually mixed in it. Subsequently, the phase two mixture was gradually poured into the phase one mixture, mixed, and maintained under constant mechanical agitation at 600 rpm until a complete mixture was achieved^39^. Additionally, experiments were conducted with different amounts of the organic phase to find a stable mixture that would interact appropriately with the chitosan solution, which had the highest concentration of *Yucca schidigera* extract. Furthermore, macroscopic observations were carried out over 72 h after the formation of nanoemulsion to analyze changes in the phases of the mixture.

### Determination of particle size and polydispersity index

The physical properties of particle size and polydispersity index (PDI) of the nano-capsules were analyzed using dynamic light scattering (DLS), employing a Malvern photometric correlation spectrometer (Zetasizer Ver. 7.11, United Kingdom)^39^.

### In vitro incubation

In vitro measurement techniques were used to evaluate the impact of the extracts on ruminal fluid samples over a given time. For this, narrow-mouth amber glass vials of 120 mL were used and sealed with rubber stoppers. In each vial, the following ingredients were added: one gram of substrate (diets with 14% and another with 18% protein inclusion, the composition of which is detailed in Table 1); variable doses of *Yucca schidigera* nano-capsules [0 (negative control) 0.25, 0.5 and 1.0 mL]; 10 mL of ruminal fluid; and 40 mL of nutrient solution. The nutrient solution contained buffer solution, resazurin, macrominerals, microminerals, and distilled water. It was prepared following the method described by Goering and Van Soest^40^. For the positive control, the same doses of empty chitosan nano-capsules were used. The animals received a 50:50 diet, composed of hay and commercial concentrated feed (Purina, Toluca, State of Mexico, Mexico) and fresh water ad libitum.

**Table 1:** Ingredients and chemical composition of diets used as substrates with different levels of proteins and characterization of Chitosan + *Yucca schidigera* nanoparticles.

	Protein levels (%)	
	14	18
Ingredients (%)		
Alfalfa hay	9.1	8.1
Wheat grains	25.0	20.0
Corn grains	25.0	27.0
Bran	13.9	14.0
Corn gluten	12.9	12.4
Soyabean meal	2.0	12.4
Molasses	12.0	6.0
Vit/Min	0.1	0.1
Composition (%)		
Crude protein	14.66	18.66
Ether extract	18.03	17.14
Acid detergent fiber	9.46	10.76
*Neutral detergent fiber*	24.51	31.89
Nitrogen free extract	66.41	58.98
Calcium (g/kg)	1.58	1.47
Phosphorus (g/kg)	3.75	4.30
Magnesium (g/kg)	1.76	1.82
Sodium (g/kg)	0.61	0.46
Potassium (g/kg)	9.47	9.35
Chloride (g/kg)	0.70	0.66
Zinc (g/kg)	22.83	20.43
Copper (g/kg)	8.19	5.83
Iron (g/kg)	123.26	99.77
Characterization of Chitosan + *Yucca schidigera* nanoparticles		
Particle size (d.nm)	St Dev (d.nm)	PDI*
244.8	15.85	0.212

*Polydispersity index.

A total of 288 bottles (3 bottles of each triplicate sample within each one of the 2 protein levels (14% and 18%) with 4 different extract types (negative control (without extract), positive control (Nano-chitosan), crude extract of *Y. schidigera*, nano-capsules of *Y. schidigera* extract) of 4 extract doses (0-, 0.25-, 0.5- and 1- mL) in 3 runs on different weeks, with 3 bottles as blanks (i.e., rumen fluid only), were incubated for 48 h. Once all bottles had been filled, they were immediately closed with rubber stoppers, shaken, and placed in the incubator at 39 ºC. The volume of gas produced, methane, carbon monoxide, and hydrogen sulfide production were recorded at 2, 4, 6, 24, 28, 30, and 48 h of inoculation.

**Fig. 1:**
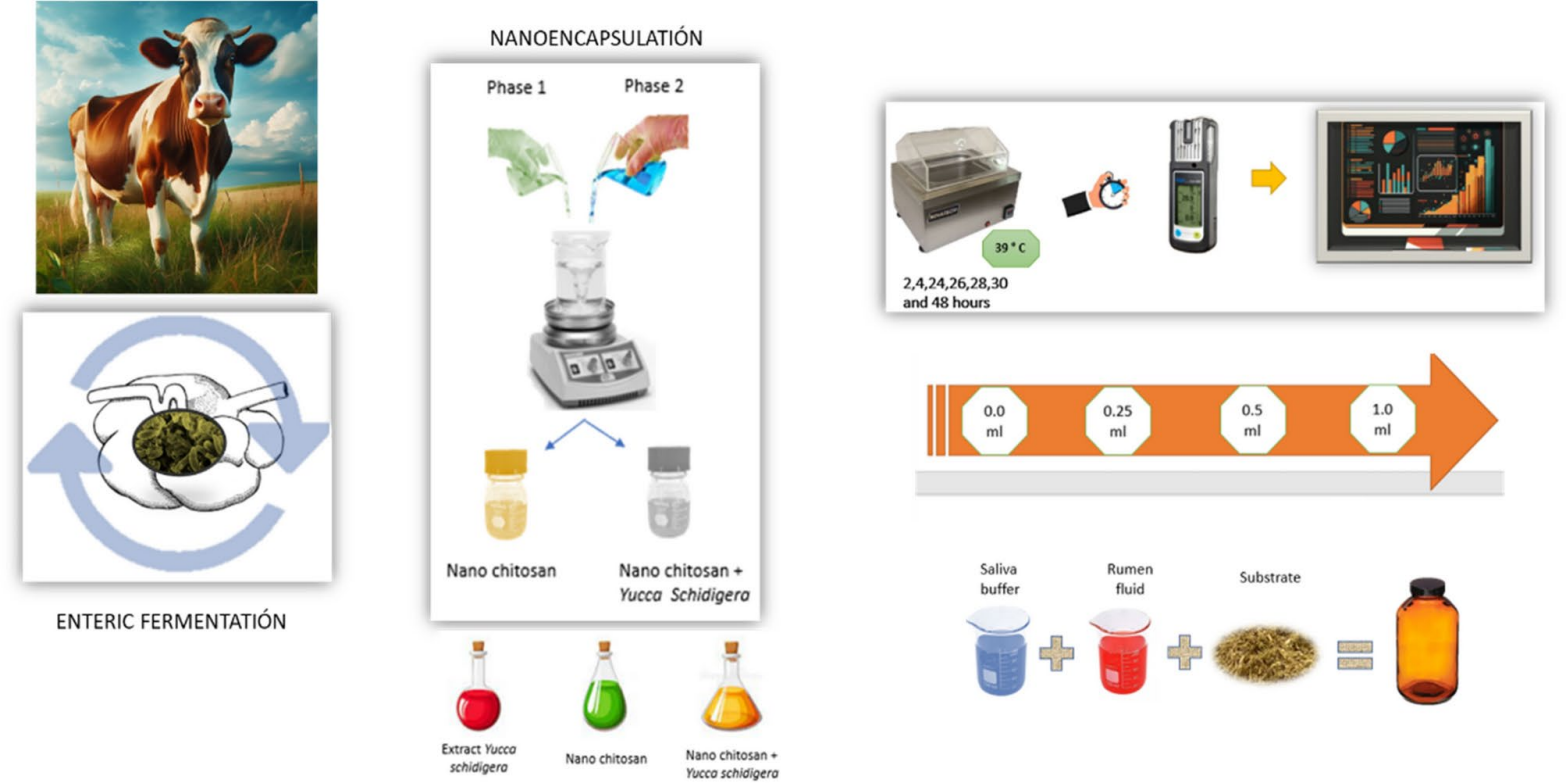
Methodology flowchart that summarizes the experimental design provides a visual overview of the research conducted.

The ruminal fluid was obtained from four bulls (400 ± 25 kg live weight), These animals were crosses of the Brahman breed with Limousin and Brahman with Charolais, that were slaughtered at the municipal slaughterhouse in Toluca, State of Mexico, Mexico, certified by the Official Mexican Standard NOM-033-SAG/ZOO-2014, which establishes the methods for the humane slaughter of domestic and wild animals. The rumen content of each animal was collected immediately after slaughtering, transported separately in a hermetic thermos, and brought within no more than 30 min to the Bromatology Laboratory of the Autonomous University of the State of Mexico. It was then filtered through four layers of sterile cotton gauze (Model FN17100, Lab. Dibar, CDMX, Mexico) to remove coarse material, allowing only microorganisms to pass. Then, a final mixture of the four filtered ruminal fluids was made (Fig. 1).

### Ruminal total gas, CH_4_, CO and H_2_S production

Using the vials, the treatments were dosed and incubated in a water bath at a constant temperature of 39 °C for 48 h. The total gas production was measured in psi, and recordings were made at the following hours: 2, 4, 24, 26, 28, 30 and 48 h after inoculation using the methodology described by Theodorou et al.^41^. At these same time intervals, measurements of CH_4_, CO and H_2_S were made, using a 5 mL volume of gas in a diffusion gas detector (Dräger Safety X-am 20500 MONITOR, Lübeck, Germany). After each measurement, the gas from each bottle was dispersed to avoid accumulation and maintain a constant gas pressure of 48 kPa in the space. Three replicates were performed for each of the treatments in each incubation cycle, three blank negative controls (without substrate) per inoculum, as well as the chitosan (same extract doses) positive control, to make a correction of readings and reduce interference of external errors in the data.

### Ruminal pH and dry matter degradability

After the incubation had been completed, the contents of the vials were filtered through filter bags with a pore size of 25 μm (F57 filter bags, ANKOM Technology Corp., Macedonia, NY, USA), separating the liquid from the undegraded part of the diet in beakers. The pH was measured with a potentiometer (HALO HI11102 wireless pH electrode, Hanna Instruments, Woonsocket, RI, USA). The undegraded diet material that remained in the filter bags was washed with abundant tap water and then maintained at 60 °C for 48 h to achieve dehydration and obtain its dry weight, a value used to obtain degradability.

### Chemical analysis

A proximate analysis of the diet samples was performed following the procedures described in^42^, where 3 subsamples of each were taken. For the fiber fraction, an ANKOM200 fiber analyzer unit (ANKOM Technology Corp., Macedon, NY, USA) was used. The determination of the neutral detergent fiber (NDF) and acid detergent fiber (ADF) fractions were determined following the procedure described according to Van Soest et al.^43^.

### Calculations and statistical analysis

For the statistical analysis, a completely randomized experimental design with a factorial arrangement (2 × 4 × 4) with three replications was used, where the values of each incubation run were averaged and used as the experimental unit for each treatment. Factor 1 was the dietary protein level (14- and 18- %), factor 2 was the types of extracts used (negative control (without extract), positive control (empty chitosan nano-capsules), *Y. schidigera* extract nano-capsules, and crude *Y. schidigera* extract), and factor 3 was the dose of each type of extract (0-, 0.25-, 0.5-, and 1- mL extract/g DM).

To obtain the values of asymptotic production, as well as to evaluate the production rate and lag phase time of each gas, the NLIN procedure of the Statistical Analysis System^44^ and the model proposed by France et al.^44^ were used, using the measurements obtained from the production volumes (mL/g DM incubated) of total biogas, CH_4_, CO, and H_2_S. To obtain the value of short-chain fatty acids (SCFA; mmol/200 mg DM), they were estimated according to Getachew et al.^45^ While the value of metabolizable energy (ME; MJ/kg DM) was estimated using the equation proposed by Getachew et al.^46^. Likewise, the CH_4_ conversion efficiency was evaluated through the production of CH_4_ per unit of SCFA (CH_4_: SCFA), ME (CH_4_: ME), and MO (CH_4_: MO) in mmol/mmol, g/MJ, and mL/g, respectively.

Data analysis was performed using the GLM procedure of SAS^47^ and the statistical model listed below:

$${\text{Y}}_{{{\text{ijk}}}} = {\upmu } + {\text{CH}}_{{\text{i}}} + {\text{TE}}_{{\text{j}}} + {\text{EX}}_{{\text{k}}} + \left( {{\text{CH}} \times {\text{TE}}} \right)_{{{\text{ij}}}} + \left( {{\text{CH}} \times {\text{EX}}} \right)_{{{\text{ik}}}} + \left( {{\text{TE}} \times {\text{EX}}} \right)_{{{\text{jk}}}} + \left( {{\text{CH}} \times {\text{TE}} \times {\text{EX}}} \right)_{{{\text{ijk}}}} + {\upvarepsilon }_{{{\text{ijk}}}}$$

Where, Y_ijk_ is the response variable, μ is the general mean, CH_i_ is the effect of the dietary protein level, TE_j_ is the effect of the type of extract, EX_k_ is the effect of the extract doses, (CH × TE)_ij_ is the effect of the interaction between the protein level and the type of extract, (CH × EX)_ik_ is the effect of the interaction between the protein level and the extract doses, (TE × EX)_jk_ is the effect of the interaction between the type of extract and the extract doses, (CH × TE × EX)_ijk_ is the effect of the interaction between the protein level, the type of extract and the extract doses, and ε_ijk_ is the experimental error. To analyze the values statistically, a comparison of means was performed using Tukey’s test, and differences were considered significant when the *p*-value was ≤ 0.05.

## Results

### Ingredients and chemical composition of diets used; and characterization of Chitosan + *Y. schidigera* nanoparticles

Results of the ingredients and chemical composition of diets used as substrates with different levels of proteins (Table 1) showed a 4% difference in the protein levels used. The Chitosan + *Yucca schidigera* nanoparticles were characterized to be 244.8 ± 15.85 (d.nm) in particle size with a polydispersity index of 0.212.

### Effect on ruminal total gas production

The results of the effect of nanoparticles of *Y. schidigera* at different doses of each extract (mL of extract/g dietary DM) on ruminal total gas production of diets with two dietary levels of protein (14- and 18- %) compared with nanoparticles of chitosan (as a positive control) and the crude extract using male bulls as a source of ruminal inoculum is as presented in Table 2. The third order interaction, PRO × TE × ED, was significant for b (*p* = 0.035), lag (*p* = 0.002), and the volume of gas produced at 48 h (*p* = 0.038) while the c, 6 h, and 24 h gas production could not be altered. The PRO × TE affected gas produced at 6 h (*p* = 0.009) while the TE × ED and PRO × ED interactions did not affect the parameters assessed. The type of extract, TE, affected all parameters under consideration except for c, while ED influenced lag (*p* = 0.011) and gas production at 6 h (*p* = 0.017). Although the interaction between TE and ED was not affected at 14% protein level, TE significantly affected b (*p* = 0.002), lag (*p* = 0.0155), 6 h (*p* =  < 0.0001), and 48 h (*p* = 0.031) while only the lag was influenced by ED (*p* = 0.024). At the higher protein level, the interaction between TE and ED significantly affected c (*p* = 0.002) and lag (*p* = 0.001). The TE affected all the gas production parameters under consideration while the effect of ED was limited to c (*p* = 0.004) and the volume of gas produced at 6 h and 24 h (*p* = 0.008 and *p* = 0.004, respectively). There was a similarity in the value of ‘c’ when 0.5 mL nano extract and 0.5 mL crude extract were used, these were, however, higher than the value obtained in the use of crude extract at 0.25 mL inclusion. The lag for the use of 0.25 mL nano chitosan was lower than the value obtained when 0.25 nano extract was used.

**Table 2:** Effect of nanoparticles of *Y. schidigera* at different doses of each extract (ml of extract /g dietary DM) on ruminal total gas production (ml/g DM) of diets with two dietary levels of protein compared with nanoparticles of chitosan (as positive control (PC)) and the crude extract using male bulls as a source of ruminal inoculum.

Protein level (PRO, % of diet)	Type of extract (TE)	Extract dose (ED; mL/g DM)	Gas production					
			Gas production kinetics^a^			mL GP g^−1^ DM incubated		
			*b*	*c*	*Lag*	6 h	24 h	48 h
14	Without extract	0.00	242.57	0.0288	1.27	64.30	129.93	213.63
	Nano-chitosan	0.25	337.33	0.0288	1.38	80.90	205.67	322.00
		0.50	408.62	0.0276	2.89	89.00	264.43	432.10
		1.00	378.30	0.0300	2.30	79.07	223.00	361.70
	Crude extract	0.25	539.28	0.0213	0.36	114.17	249.40	518.17
		0.50	472.07	0.0279	2.41	96.97	221.73	436.80
		1.00	481.57	0.0281	2.01	132.40	290.20	456.47
	Nano-extract	0.25	401.45	0.0296	2.46	75.93	209.73	377.80
		0.50	425.02	0.0513	2.84	70.63	202.07	407.83
		1.00	488.08	0.0312	5.42	80.27	218.47	456.50
		SEM^b^	35.892	0.00703	0.774	8.881	21.946	42.023
		TE	0.0018	0.1385	0.0155	< .0001	0.0740	0.0310
		ED	0.7294	0.2999	0.0244	0.2898	0.4646	0.8124
		TE × ED	0.2061	0.4248	0.2776	0.1996	0.1589	0.1824
18	Without extract	0.00	464.27	0.0292	3.98	81.87	212.77	432.00
	Nano-chitosan	0.25	339.83	0.0275	0.89	81.93	197.93	321.73
		0.50	335.60	0.0269	1.25	82.77	195.90	317.53
		1.00	352.97	0.0269	2.60	96.90	218.23	334.97
	Crude extract	0.25	445.68	0.0268	0.94	99.20	2296.67	414.50
		0.50	500.20	0.0305	4.53	88.60	228.57	466.10
		1.00	507.33	0.0291	3.05	101.77	245.37	472.93
	Nano-extract	0.25	415.55	0.0291	4.73	76.83	204.83	388.53
		0.50	455.85	0.0305	4.29	79.30	213.13	426.40
		1.00	416.20	0.0275	1.44	88.37	222.70	390.17
		SEM^b^	16.294	0.00055	0.653	4.473	6.519	14.367
		TE	< .0001	0.0006	0.0063	0.002	< .0001	< .0001
		ED	0.0751	0.0038	0.0848	0.0082	0.0039	0.0519
		TE × ED	0.1642	0.0022	0.001	0.5252	0.8917	0.1241
Pooled SEM^2^			27.872	0.00499	0.716	7.031	16.188	31.403
*p*-value								
PRO			0.0228	0.4599	0.0447	0.6492	0.4809	0.1136
TE			< .0001	0.0936	0.0007	< .0001	0.0033	< .0001
ED			0.2889	0.1832	0.0107	0.0168	0.0846	0.3540
PRO × TE			0.7511	0.1534	0.0808	0.0085	0.2430	0.6001
PRO × ED			0.7719	0.4294	0.0960	0.9342	0.9625	0.9702
TE × ED			0.7734	0.3067	0.0688	0.2256	0.2134	0.5838
PRO × TE × ED			0.0349	0.5087	0.0020	0.2226	0.1261	0.0378

^a^*b* = asymptotic gas production (mL g^−1^ DM); *c* = rate of gas production (mL h^−1^); *Lag* = initial delay before gas production begins (h).

^b^ SEM = standard error of the mean. GP – Gas Production.

**Fig. 2:**
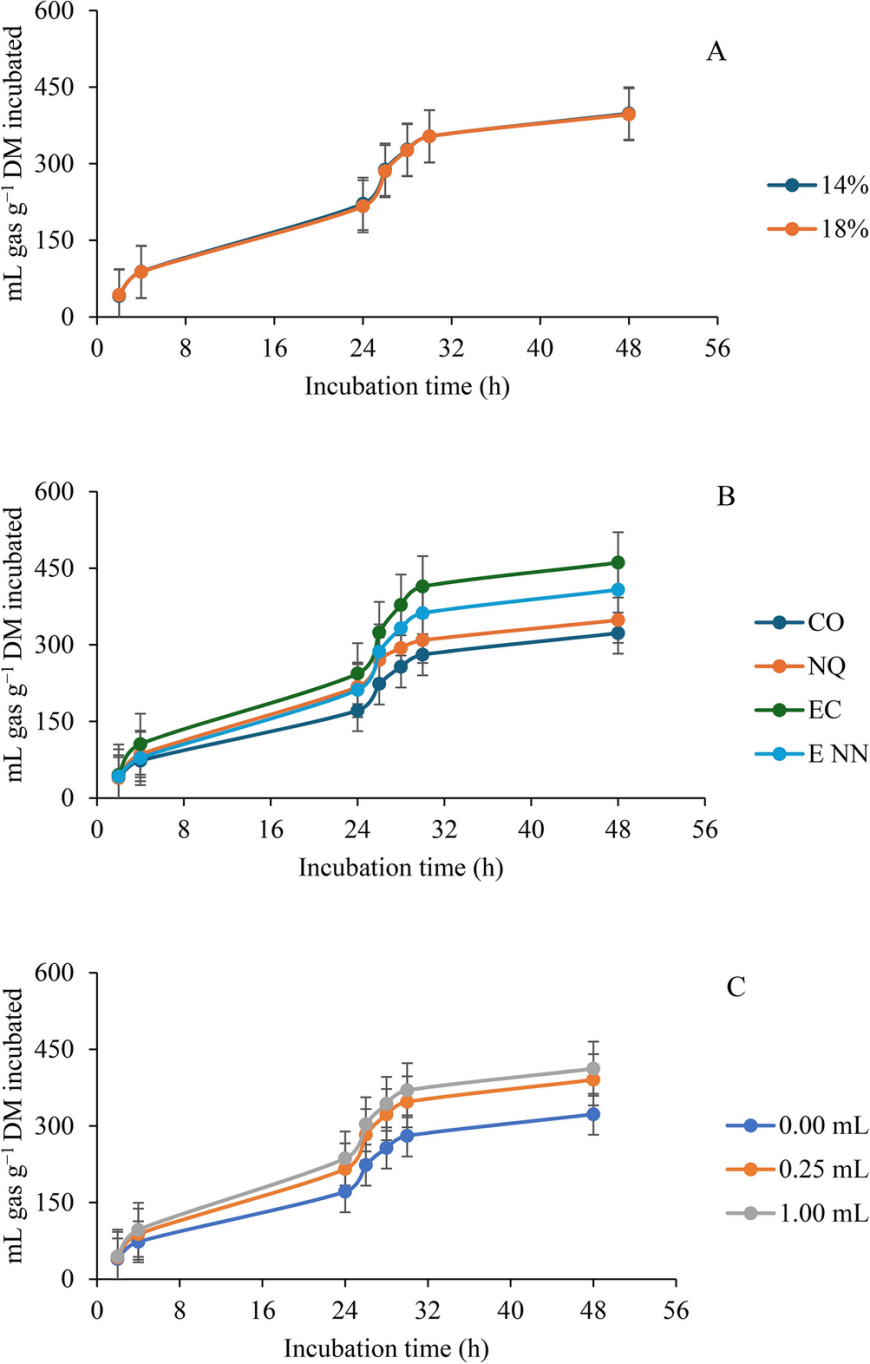
Total gas production during the in vitro rumen fermentation response to (**A**) percentage of protein in the diet, (**B**) extract type, and (**C**) extract dose. The bars indicate the standard error of the mean. CO: Control; NQ: Nano-chitosan; EC: Crude extract; ENN: Nano-extract.

Figure 2A–C showed similarity in the rate of gas production between crude protein levels of 14 and 18%, the pattern/rate appeared the same regardless of ED and TE. Gas production initiation started at about 2 h of incubation, increased to 4 h, from where it gained momentum steadily to 24 h, where there was a sudden upsurge to 30 h, and then gradually halted to 48 h. The control generated more gas than other TE while ED of 1.0 produced more gas as well.

### Effect on ruminal methane production

The effect of nanoparticles of *Y. schidigera* at different doses of each extract (mL of extract/g dietary DM) on ruminal methane production (CH_4_, mL/g DM) of diets with two dietary levels of protein compared with nanoparticles of chitosan (as positive control) and the crude extract using male bulls as a source of ruminal inoculum is as shown on Table 3. The PRO × TE × ED interaction significantly affected CH_4_ production (mL CH_4_ g/DM incubated) only at 24 h (*p* = 0.045), TE × ED influenced most of the parameters (b, 6 h, 24 h, 48 h mL CH_4_ g/DM, 6 h and 24 h mL CH_4_ 100 mL/GP at *p* = 0.037, 0.001, 0.004, 0.037, 0.01 and 0.041 respectively) except c, lag, and 48 h. PRO × ED did not affect all the parameters under consideration while PRO × TE affected all the parameters assessed except c. TE affected all except CH_4_ (mL CH_4_/100 mL GP) produced at 24 h, and ED only affected CH_4_ (mL CH_4_/100 mL GP) production at 6 h (*p* = 0.024).

**Table 3:** Effect of nanoparticles of *Y. schidigera* at different doses of each extract (ml of extract /g dietary DM) on ruminal methane production (CH_4_, ml/g DM) of diets with two dietary levels of protein compared with nano-particles of chitosan (as a positive control (PC) and the crude extract using male bulls as a source of ruminal inoculum.

Protein level (PRO, % of diet)	Type of extract (TE)	Extract dose (ED; mL/g DM)	CH_4_ production								
			CH_4_ production kinetics^a^			mL CH_4_ g^−1^ DM incubated			mL CH_4_ 100 mL^−1^ GP		
			*b*	*c*	*Lag*	6 h	24 h	48 h	6 h	24 h	48 h
14	Without extract	0.00	33.50	0.1230	3.79	0.55	3.74	33.83	0.87	3.03	16.87
	Nano-chitosan	0.25	57.13	0.0923	3.63	0.91	9.74	57.14	1.25	5.08	19.51
		0.50	126.57	0.0897	3.22	1.59	19.37	126.74	2.02	8.41	33.55
		1.00	72.83	0.1125	3.72	0.60	5.45	72.84	0.72	2.44	20.10
	Crude extract	0.25	113.37	0.1134	3.79	2.37	11.96	113.34	2.10	4.77	21.60
		0.50	83.80	0.1029	3.95	1.40	9.04	83.63	1.43	4.10	19.27
		1.00	59.07	0.0940	2.53	1.49	12.05	58.87	1.13	4.13	12.97
	Nano-extract	0.25	91.30	0.1293	3.95	0.73	9.72	91.62	0.97	4.63	24.30
		0.50	88.23	0.1062	3.77	0.99	6.21	88.66	1.40	3.07	21.57
		1.00	119.03	0.1213	4.11	1.08	8.73	119.31	1.30	3.97	25.80
		SEM^b^	22.680	0.01051	0.601	0.252	2.345	22.684	0.344	1.346	6.728
		TE	0.6858	0.0640	0.538	0.0013	0.2037	0.6723	0.4907	0.4326	0.4418
		ED	0.6727	0.3505	0.7922	0.3311	0.3571	0.6696	0.1444	0.2965	0.6459
		TE × ED	0.1209	0.3416	0.5045	0.0159	0.0082	0.1200	0.1338	0.1282	0.5733
18	Without extract	0.00	120.37	0.0900	4.65	0.59	9.33	120.27	0.73	4.40	27.90
	Nano-chitosan	0.25	15.80	0.0930	2.08	0.45	3.31	15.81	0.53	1.65	4.84
		0.50	16.40	0.0959	2.51	0.59	3.05	16.39	0.70	1.53	5.15
		1.00	18.63	0.0928	1.71	0.97	4.36	18.65	1.00	2.00	5.58
	Crude extract	0.25	100.17	0.0921	3.57	2.52	15.93	99.51	2.50	7.00	23.83
		0.50	66.70	0.1214	3.96	1.57	9.33	66.47	1.77	4.10	14.27
		1.00	64.70	0.1272	4.06	1.03	9.01	64.55	1.00	3.67	13.50
	Nano-extract	0.25	139.93	0.1680	5.15	1.23	12.33	138.65	1.60	6.10	35.77
		0.50	113.80	0.1310	4.70	1.62	7.17	113.79	2.03	3.37	26.70
		1.00	119.53	0.1131	4.08	1.31	14.09	119.41	1.50	6.33	30.50
		SEM^2^	9.759	0.01583	0.490	0.254	1.914	9.701	0.235	0.856	2.124
		TE	< .0001	0.0111	< .0001	0.0002	< .0001	< .0001	< .0001	< .0001	< .0001
		ED	0.0432	0.8646	0.5419	0.3715	0.0540	0.0491	0.1244	0.0412	0.0050
		TE × ED	0.3258	0.1049	0.5718	0.0073	0.1041	0.3381	0.0050	0.0988	0.0764
Pooled SEM^b^			17.459	0.01343	0.548	0.253	2.141	17.445	0.294	1.128	4.989
*p*-value											
PRO			0.6191	0.9547	0.6994	0.8703	0.9544	0.6507	0.9972	0.7859	0.6364
TE			< .0001	0.0007	0.0002	< .0001	0.0178	< .0001	0.0025	0.1698	0.0002
ED			0.5670	0.6714	0.5115	0.1370	0.3789	0.5783	0.0243	0.2263	0.4692
PRO × TE			0.0002	0.3436	0.0031	0.0260	0.0002	0.0002	0.0089	0.0009	0.0002
PRO × ED			0.2945	0.6236	0.8996	0.8882	0.0602	0.3046	0.7424	0.0980	0.2810
TE × ED			0.0365	0.1341	0.9201	0.0005	0.0035	0.0367	0.0096	0.0401	0.2111
PRO × TE × ED			0.3866	0.1251	0.2515	0.0614	0.0448	0.3831	0.0910	0.2424	0.8951

^a^*b* = asymptotic CH_4_ production (mL g^−1^ DM); *c* = rate of CH_4_ production (mL h^−1^); *Lag* = initial delay before CH_4_ production begins (h).

^b^SEM = standard error of the mean.

At the lower crude protein level, the interaction between TE and ED showed a significant difference in the volume of CH_4_ produced at 6 h (*p* = 0.016) and 24 h (*p* = 0.008). Although ED did not affect CH_4_ production kinetics, however, TE significantly (*p* = 0.001) affected it at 6 h. Using crude extract at an ED of 0.25 more CH_4_ was produced at 6 h but with less generated without the use of extract. Nano-chitosan at 0.05 mL generated more CH_4_ than the 3.74 produced without extract at 24 h. When a crude protein level of 18% was used, TE and ED interaction significantly affected CH_4_ production (mL CH_4_/g DM incubated; mL CH_4_/100 mL GP) at 6 h (*p* = 0.007) and 6 h (*p* = 0.005). TE significantly affected all the parameters under consideration but with ED influencing b (*p* = 0.043), CH_4_ production (mL CH_4_/g DM incubated) at 48 h (*p* = 0.049), and production of CH_4_ (mL CH_4_/100 mL GP) at 24 h and 48 h (*p* = 0.041 and 0.005 respectively). The use of crude extract at 0.25 mL/g DM elicited higher gas production at 6 h but with lower gas produced when nano chitosan was used at the same extract dose.

**Fig. 3:**
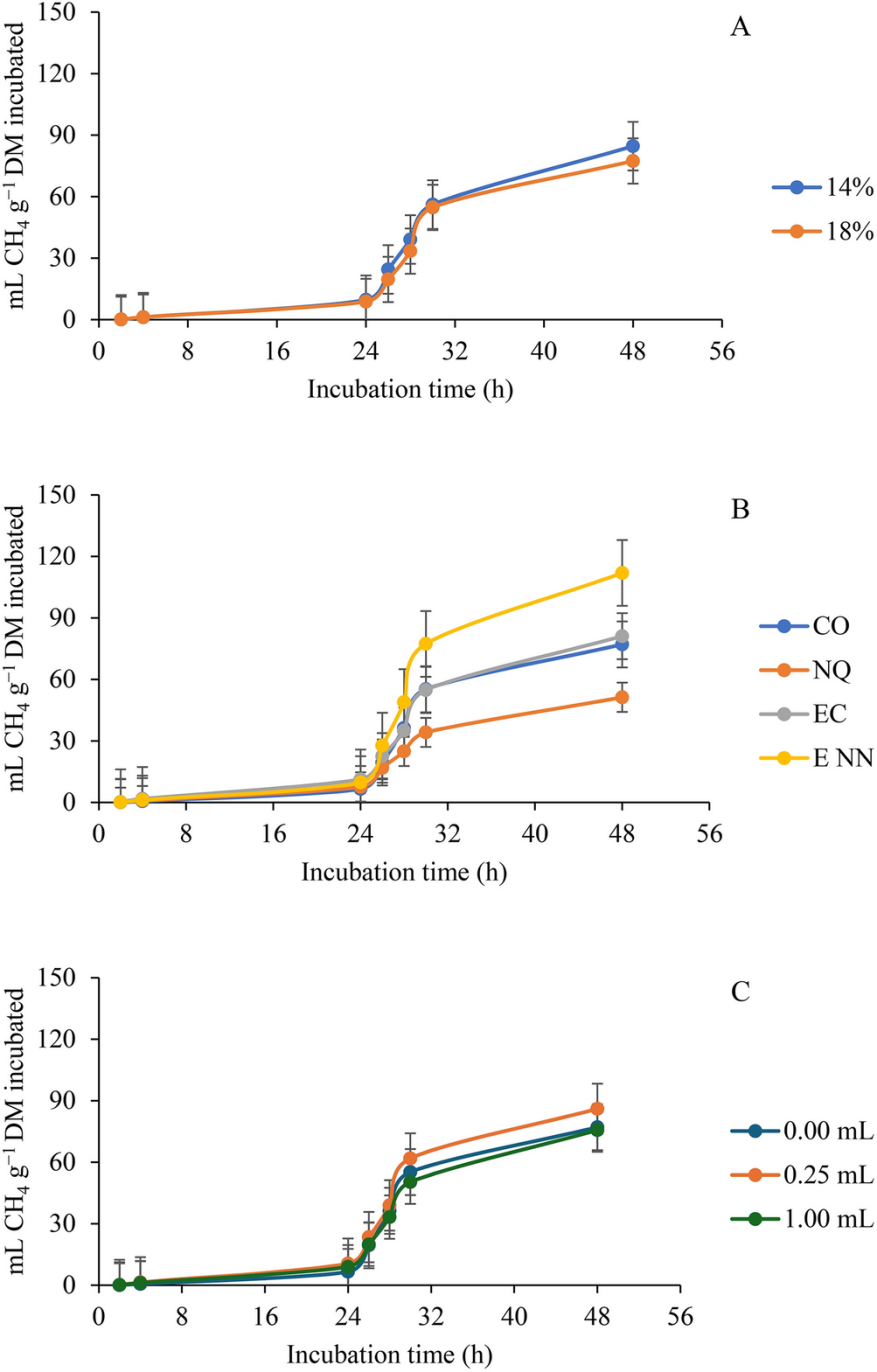
Total methane (CH_4_) production during the in vitro ruminal fermentation response to (**A**) percentage of protein in the diet, (**B**) extract type, and (**C**) extract dose. The bars indicate the standard error of the mean. CO: Control; NQ: Nano-chitosan; EC: Crude extract; ENN: Nano-extract.

Methane production (mL CH_4_/g DM incubated) accompanied gas production (Fig. 3A), and this followed the pattern of gas production except that it appeared lower protein in the diet was associated with more methane gas production. This was observed 24 h after the initiation of incubation, and more CH_4_ was generated by nano-extract and at the ED of 0.25 mL/g DM than others (Fig. 3B and C).

### Effect on ruminal carbon monoxide

Effect of nanoparticles of *Y. schidigera* at different doses of each extract (0-, 0.25-, 0.5-, and 1- mL of extract/g dietary DM) on ruminal carbon monoxide of diets with two dietary levels of protein (14- and 18- %) compared with nanoparticles of chitosan (as positive control) and the crude extract using male bulls as a source of ruminal inoculum is as presented in Table 4. The results observed were that the interaction among PRO, TE, and ED significantly (*p* = 0.034) affected c while The TE × ED had a significant effect on the CO produced at 6 and 24 h (*p* =  < 0.0001 and 0.024 respectively). The PRO × TE influenced b (*p* = 0.047) while PRO × ED and PRO did not influence the parameters assessed for CO gas. However, TE had a significant effect on CO production at 6 h and 24 h (*p* =  < 0.0001 and 0.0013 respectively) and ED was impactful at 6 h (*p* = 0.0047). At a lower protein level, c and 6 h (*p* = 0.024 and 0.001) were influenced by the interaction of both TE and ED while TE affected b (*p* = 0.048), c (*p* = 0.025), and 6 h (*p* = 0.001) significantly. The Extract Dose (ED) was effective only at 6 h (*p* = 0.047). Higher c was obtained when a 1 mL dosage of nano-extract was incubated using a lower protein level but with a lower value observed with 1 mL nano-chitosan inclusion. The CO production at 6 h was lower and higher when no extract was included and at 0.25 mL/g DM crude extract dose, respectively. At the protein level of 18%, the interaction of TE and ED significantly impacted CO production at 6 and 12 h (*p* = 0.008 and 0.0379 respectively). The TE significantly influenced CO production at 6 and 12 h (*p* = 0.019 and 0.0048) while ED was of no effect, The extract dose of 0.25 mL/g DM for both the crude extract and nano-extract elicited higher and lower CO production at 6 h, respectively. The crude extract at ED of 0.25 mL/g DM also elicited higher CO production at 24 h.

**Table 4:** Effect of nanoparticles of *Y. schidigera* at different doses of each extract (ml of extract /g dietary DM) on ruminal carbon monoxide (CO, ml/g DM) of diets with two dietary levels of protein compared with nanoparticles of chitosan (as positive control (PC)) and the crude extract using male bulls as a source of ruminal inoculum.

Protein level (PRO, % of diet)	Type of extract (TE)	Extract dose (ED; mL/g DM)	CO production					
			CO production kinetics^a^			mL CO g^−1^ DM incubated		
			*b*	*c*	*Lag*	6 h	24 h	48 h
14	Without extract	0.00	0.1101	0.00016	0.0013	0.0016	0.0083	0.0495
	Nano-chitosan	0.25	0.2775	0.00051	0.0004	0.0018	0.0108	0.1299
		0.50	0.4230	0.00022	0.0064	0.0032	0.0281	0.1946
		1.00	0.5297	0.00014	0.0013	0.0027	0.0181	0.2560
	Crude extract	0.25	0.3547	0.00026	0.0035	0.0082	0.0298	0.1639
		0.50	0.1950	0.00040	0.0008	0.0021	0.0141	0.0936
		1.00	0.4418	0.00056	0.0014	0.0031	0.0386	0.2121
	Nano-extract	0.25	0.1760	0.00050	0.0007	0.0015	0.0127	0.0827
		0.50	0.2201	0.00042	0.0007	0.0017	0.0150	0.0992
		1.00	0.2713	0.00064	0.0007	0.0013	0.0145	0.1212
		SEM^b^	0.08672	0.000095	0.00217	0.00077	0.00704	0.04358
		TE	0.0484	0.0251	0.5412	0.0005	0.0852	0.0527
		ED	0.0983	0.4032	0.6755	0.0469	0.5607	0.1056
		TE × ED	0.4548	0.0237	0.3734	0.0006	0.128	0.5274
18	Without extract	0.00	0.2961	0.00010	0.0010	0.0034	0.0187	0.1350
	Nano-chitosan	0.25	0.1863	0.00043	0.0009	0.0022	0.0120	0.0824
		0.50	0.2316	0.00027	0.0007	0.0026	0.0137	0.1046
		1.00	0.2802	0.00046	0.0004	0.0022	0.0098	0.1286
	Crude extract	0.25	0.4060	0.00045	0.0066	0.0086	0.0422	0.1920
		0.50	0.2747	0.00020	0.0006	0.0024	0.0192	0.1305
		1.00	0.2370	0.00021	0.0016	0.0019	0.0168	0.1061
	Nano-extract	0.25	0.2267	0.00045	0.0022	0.0012	0.0111	0.0956
		0.50	0.2562	0.00035	0.0005	0.0017	0.0152	0.1208
		1.00	0.2428	0.00019	0.0011	0.0021	0.0154	0.1130
		SEM^b^	0.04691	0.000128	0.00198	0.00108	0.00503	0.02343
		TE	0.1405	0.6300	0.3794	0.0196	0.0048	0.1257
		ED	0.8465	0.2198	0.2402	0.0793	0.1721	0.9262
		TE × ED	0.0994	0.6339	0.6320	0.0084	0.0379	0.0857
Pooled SEM^b^			0.06972	0.000113	0.00208	0.00094	0.00612	0.03498
*p*-value								
PRO			0.7637	0.1995	0.8530	0.4696	0.9483	0.6718
TE			0.0532	0.3501	0.4910	< .0001	0.0013	0.0524
ED			0.1895	0.1725	0.5574	0.0047	0.8187	0.2001
PRO × TE			0.0474	0.0796	0.4183	0.9227	0.5757	0.0529
PRO × ED			0.1054	0.3958	0.3020	0.9147	0.1645	0.1164
TE × ED			0.1129	0.7525	0.1995	< .0001	0.0237	0.1495
PRO × TE × ED			0.7411	0.0340	0.9114	0.8497	0.1833	0.7000

^a^*b* = asymptotic CO production (mL g^−1^ DM); *c* = rate of CO production (mL h^−1^); *Lag* = initial delay before CO production begins (h).

^b^SEM = standard error of the mean.

**Fig. 4:**
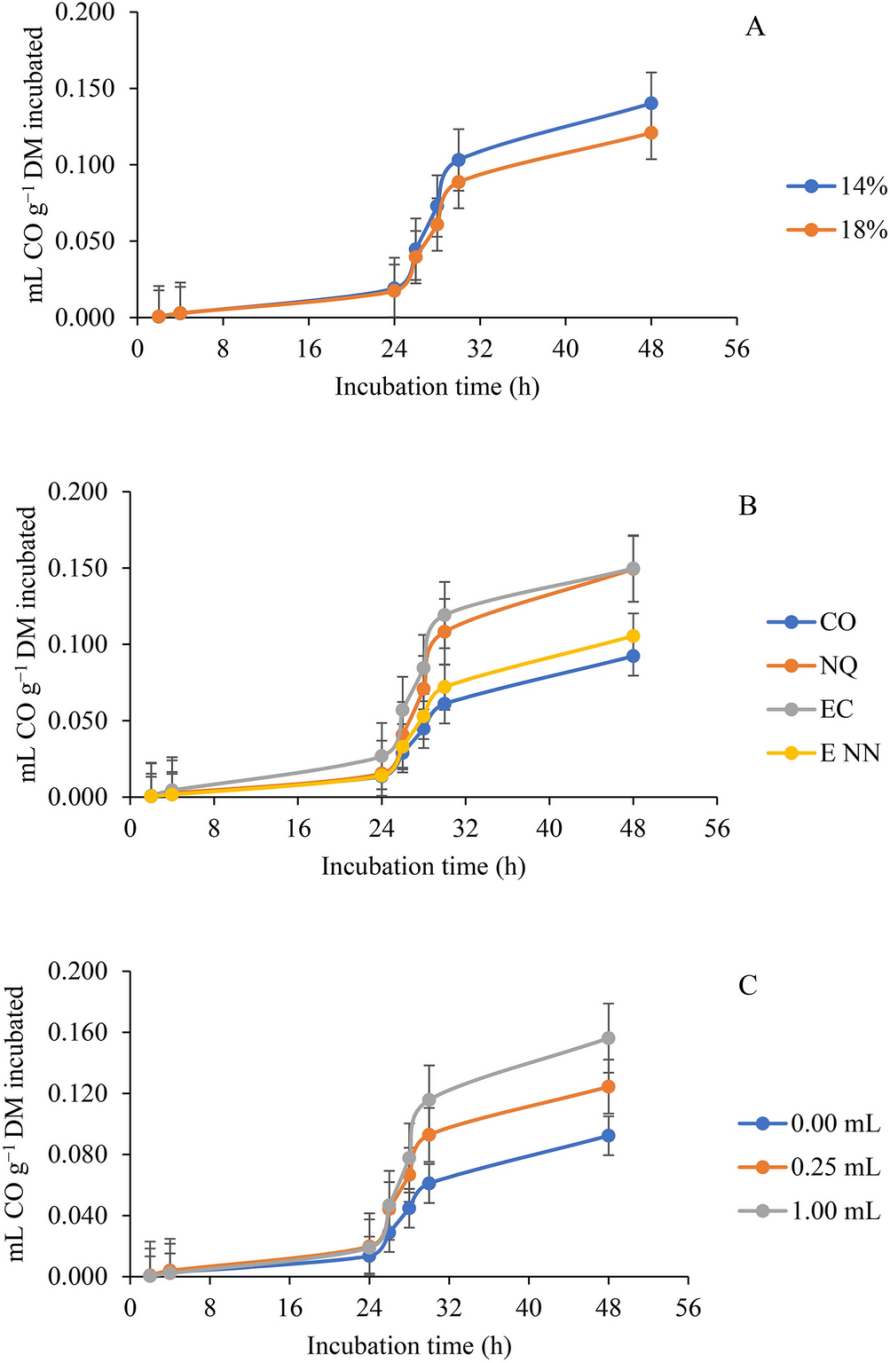
Total carbon monoxide (CO) production during in vitro ruminal fermentation response to (**A**) percentage of protein in the diet, (**B**) extract type, and (**C**) extract dose. The bars indicate the standard error of the mean. CO: Control; NQ: Nano-chitosan; EC: Crude extract; ENN: Nano-extract.

More CO was produced at a crude protein level of 14%, by nano-chitosan and control at 48 h, and at 1 mL compared to the other crude protein level, TE, and ED, respectively (Fig. 4A–C).

### Effect on ruminal hydrogen sulfide

**Fig. 5:**
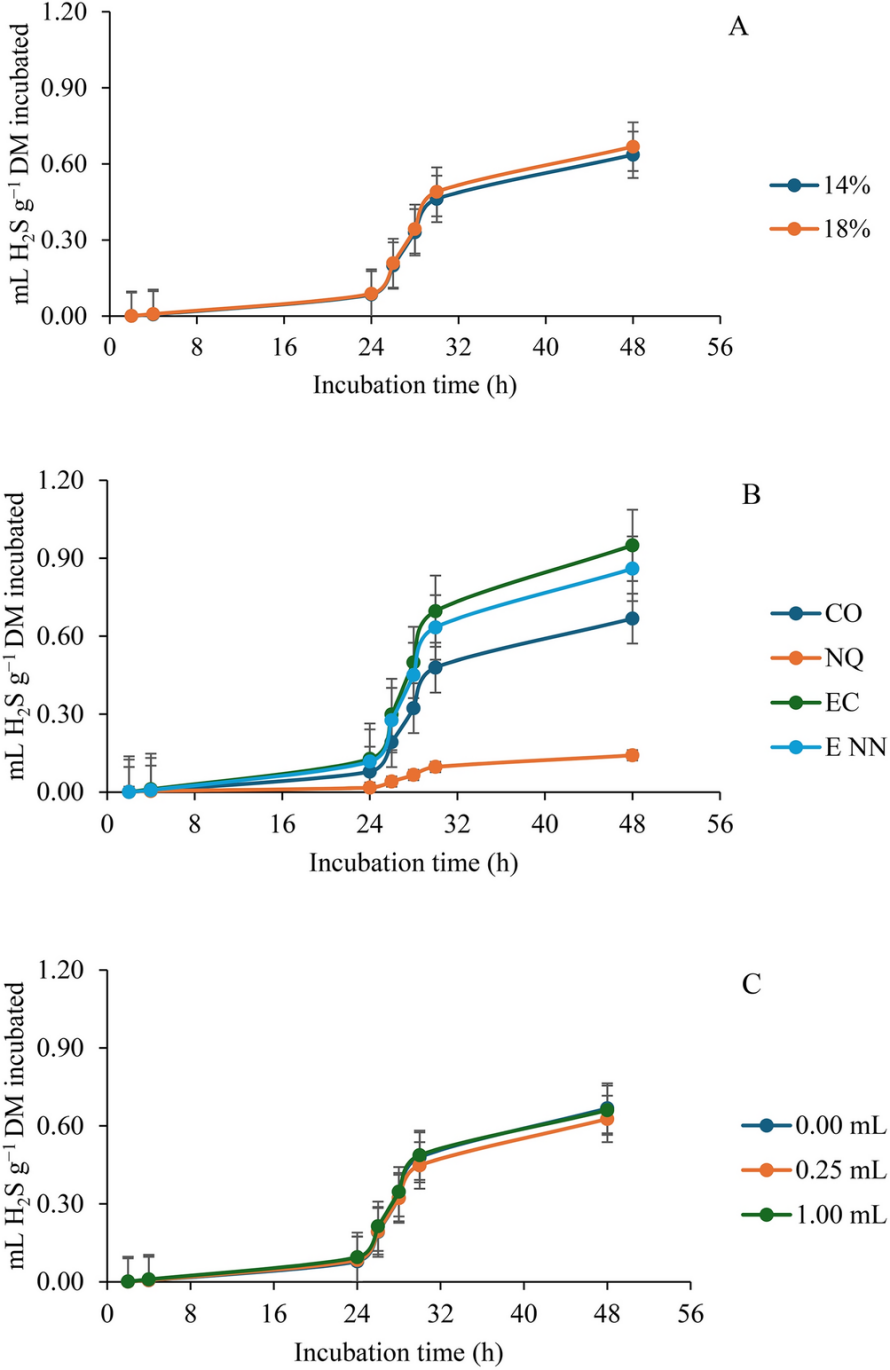
Total hydrogen sulfide (H_2_S) production during in vitro rumen fermentation in response to (**A**) percentage of protein in the diet, (**B**) extract type, and (**C**) extract dose. The bars indicate the standard error of the mean. CO: Control; NQ: Nano-chitosan; EC: Crude extract; ENN: Nano-extract.

Table 5 represents the effect of nanoparticles of *Y. Schidigera* at different doses of each extract (0-, 0.25-, 0.5-, and 1- mL of extract/g dietary DM) on ruminal hydrogen sulfide of diets with two dietary levels of protein (14- and 18- %) compared with nanoparticles of chitosan (as positive control) and the crude extract using male bulls as a source of ruminal inoculum. From the results, the PRO × TE × ED interaction impacted H_2_S production at 6 h (*p* = 0.027) and 48 h (*p* = 0.047) while PRO × TE significantly influenced only c (*p* = 0.006). The interactions PRO × TE and TE × ED showed no influence on the parameters assessed for H2S. Generally, a significant influence was observed on b, c, 6, and 48 h (*p* = 0.017, 0.003, 0.012, and 0.009 respectively) of H_2_S production for protein levels used (*i.e.,* PRO) and on b, c, 6, 24, and 48 h (*p* =  < 0.0001 for all) for TE while ED was effective at 6 h (*p* = 0.003). At the low protein diet, the production of H_2_S was not significantly influenced by TE × ED interaction and ED. However, TE impacted significantly on b (*p* =  < 0.0001), c (*p* = 0.019), 6 (*p* =  < 0.0001), 24 (*p* =  < 0.0001), and 48 h (*p* =  < 0.0001). At a higher crude protein level of 18%, the interaction of TE and ED only influenced (*p* = 0.021) H_2_S produced at 6 h. TE significantly influenced b, c, 6, 24, and 48 h (*p* =  < 0.0001). The H_2_S production at 6 and 24 h was affected by ED (*p* = 0.001 and 0.046 respectively). Nano-chitosan at 0.25 resulted in less H_2_S produced at 6 h than when crude extract was used at the same dose. There was less production of H_2_S with 14% crude protein level, control, and ED of 0.25 mL (Fig. 5A–C).

**Table 5:** Effect of nanoparticles of *Y. Schidigera* at different doses of each extract (ml of extract /g dietary DM) on ruminal hydrogen sulfide (H_2_S, ml/g DM) of diets with two dietary levels of protein compared with nanoparticles of chitosan (as positive control (PC)) and the crude extract using male bulls as a source of ruminal inoculum.

Protein level (PRO, % of diet)	Type of extract (TE)	Extract dose (ED; mL/g DM)	H_2_S production					
			H2S production kinetics^a^			mL H_2_S g^−1^ DM incubated		
			*b*	*c*	*Lag*	6 h	24 h	48 h
14	Without extract	0.00	0.9430	0.00058	0.0053	0.0036	0.0623	0.4558
	Nano-chitosan	0.25	0.2925	0.00002	0.0009	0.0045	0.0201	0.1384
		0.50	0.2848	0.00005	0.0006	0.0029	0.0146	0.1359
		1.00	0.2356	0.00001	0.0017	0.0026	0.0113	0.0997
	Crude extract	0.25	1.7626	0.00162	0.0002	0.0106	0.1311	0.9919
		0.50	1.9377	0.00076	0.0002	0.0091	0.1242	0.9449
		1.00	1.8431	0.00067	0.0023	0.0142	0.1443	0.9130
	Nano-extract	0.25	1.6798	0.00138	0.0005	0.0049	0.1079	0.8131
		0.50	1.7569	0.00032	0.0007	0.0071	0.1103	0.8614
		1.00	2.0348	0.00027	0.0003	0.0078	0.1256	1.0064
		SEM^b^	0.17897	0.000392	0.00146	0.00163	0.01212	0.07414
		TE	< .0001	0.0188	0.8865	< .0001	< .0001	< .0001
		ED	0.6864	0.0800	0.6820	0.3606	0.5504	0.8895
		TE × ED	0.7449	0.5705	0.9123	0.2715	0.7864	0.4076
18	Without extract	0.00	1.7879	0.00017	0.0018	0.0080	0.0957	0.8795
	Nano-chitosan	0.25	0.2907	0.00003	0.0008	0.0026	0.0111	0.1258
		0.50	0.2847	0.00004	0.0009	0.0036	0.0119	0.1286
		1.00	0.4323	0.00006	0.0003	0.0100	0.0338	0.2218
	Crude extract	0.25	1.7762	0.00276	0.0006	0.0111	0.1225	0.8752
		0.50	2.0053	0.00224	0.0000	0.0095	0.1216	1.0023
		1.00	1.9441	0.00143	0.0000	0.0109	0.1220	0.9691
	Nano-extract	0.25	1.6823	0.00205	0.0004	0.0060	0.1116	0.8164
		0.50	1.8228	0.00378	0.0006	0.0093	0.1172	0.9099
		1.00	1.5051	0.00187	0.0011	0.0105	0.1318	0.7503
		SEM^b^	0.08548	0.000557	0.00045	0.00115	0.00700	0.04132
		TE	< .0001	< .0001	0.3875	< .0001	< .0001	< .0001
		ED	0.2377	0.1663	0.9549	0.0012	0.0461	0.1121
		TE × ED	0.0839	0.2294	0.5397	0.0212	0.4366	0.0533
Pooled SEM^b^			0.14024	0.000481	0.00108	0.00141	0.00989	0.06001
*p*-value								
PRO			0.0165	0.0029	0.0763	0.0118	0.1830	0.0092
TE			< .0001	< .0001	0.8836	< .0001	< .0001	< .0001
ED			0.4130	0.0901	0.7282	0.0030	0.0917	0.5068
PRO × TE			0.3138	0.0059	0.7653	0.1437	0.2889	0.3353
PRO × ED			0.7465	0.1522	0.6723	0.3418	0.8292	0.5286
TE × ED			0.8873	0.3228	0.9730	0.3165	0.9016	0.8905
PRO × TE × ED			0.2504	0.2698	0.7696	0.0265	0.4866	0.0470

^a^*b* = is the asymptotic H_2_S production (mL g^−1^ DM); *c* = is the rate of H_2_S production (mL h^−1^); *Lag* = is the initial delay before H_2_S production begins (h); ^b^SEM = standard error of the mean.

### Effect on rumen fermentation profile and CH_4_ conversion efficiency

Table 6 presents the effect of nanoparticles of *Y. Schidigera* at different doses of each extract (0-, 0.25-, 0.5-, and 1- mL of extract/g dietary DM) on rumen fermentation profile and CH_4_ conversion efficiency of diets with two dietary levels of protein (14- and 18- %) compared with nanoparticles of chitosan (as positive control) and the crude extract using male bulls as a source of ruminal inoculum. The interaction PRO × TE × ED affected CH_4_:ME only (*p* = 0.045), while PRO × TE interaction influenced pH (*p* = 0.011) and CH_4_ conversion efficiency (CH_4_:SCFA, CH_4_:ME, and CH_4_:OM) (*p* = 0.0006, 0.0002 and 0.0009 respectively). The TE x ED interaction was effective for CH_4_ conversion efficiency (CH_4_:SCFA, CH_4_:ME, and CH_4_:OM) (*p* = 0.015, 0.004, and 0.04) while PRO x ED was not effective for all the parameters assessed. Protein level (i.e., PRO) influenced pH (*p* = 0.001) and ME (*p* = 0.001), while TE affected pH, DMD, SCFA, ME, and CH_4_:ME (*p* =  < 0.0001, < 0.0001, 0.003, 0.0033 and 0.018 respectively).

**Table 6:** Effect of nanoparticles of *Y. Schidigera* at different doses of each extract (ml of extract /g dietary DM) on rumen fermentation profile and CH_4_ conversion efficiency of diets with two dietary levels of protein (14 and 18%) compared with nanoparticles of chitosan (as positive control (PC)) and the crude extract using male bulls as a source of ruminal inoculum.

Protein level (PRO, % of diet)	Type of extract (TE)	Extract dose (ED; mL/g DM)	Ruminal fermentation characteristics^a^				CH_4_ conversion efficiency^b^		
			pH	DMD	SCFA	ME	CH_4_:SCFA	CH_4_:ME	CH_4_:OM
14	Without extract	0.00	7.45	87.60	5.75	7.16	2.42	4.20	19.88
	Nano-chitosan	0.25	6.60	57.97	9.11	8.89	5.27	10.94	33.25
		0.50	6.22	68.31	11.72	10.23	9.38	21.77	54.96
		1.00	6.48	57.95	9.88	9.28	2.72	6.13	15.95
	Crude extract	0.25	6.92	85.01	11.05	9.88	5.61	13.44	31.16
		0.50	7.05	87.61	9.82	9.25	4.54	10.15	26.81
		1.00	6.49	71.77	12.86	10.81	5.16	13.54	27.01
	Nano-extract	0.25	7.44	88.59	9.29	8.98	5.03	10.92	30.30
		0.50	7.37	87.73	8.95	8.81	3.27	6.97	20.06
		1.00	7.32	87.26	9.68	9.18	4.41	9.81	25.94
		SEM^b^	0.160	6.563	0.975	0.500	1.327	2.635	8.805
		TE	< .0001	0.0002	0.0745	0.0744	0.3745	0.2036	0.4325
		ED	0.2509	0.2746	0.4658	0.4635	0.3159	0.3573	0.2968
		TE × ED	0.1818	0.6893	0.1585	0.1592	0.0485	0.0081	0.129
18	Without extract	0.00	7.45	90.98	9.43	9.55	4.55	10.48	28.77
	Nano-chitosan	0.25	6.87	75.53	8.77	9.21	1.66	3.72	10.75
		0.50	7.03	63.85	8.68	9.16	1.53	3.43	10.03
		1.00	6.89	72.19	9.67	9.67	2.10	4.90	13.08
	Crude extract	0.25	7.24	90.24	10.04	9.86	7.49	17.90	45.77
		0.50	7.06	83.01	10.13	9.91	4.39	10.49	26.81
		1.00	6.94	81.80	10.87	10.29	4.07	10.12	23.97
	Nano-extract	0.25	7.45	90.19	9.07	9.36	6.16	13.85	39.89
		0.50	7.41	89.11	9.44	9.56	3.49	8.06	22.02
		1.00	7.39	88.47	9.87	9.77	6.71	15.84	41.41
		SEM^b^	0.077	5.610	0.289	0.148	0.905	2.150	5.597
		TE	< .0001	0.0015	< .0001	< .0001	< .0001	< .0001	< .0001
		ED	0.1970	0.3519	0.0039	0.0039	0.0467	0.0539	0.0412
		TE × ED	0.1656	0.8292	0.8922	0.8857	0.0988	0.1037	0.0986
Pooled SEM^c^			0.125	6.105	0.719	0.369	1.136	2.405	7.377
*p*-value									
PRO			0.0010	0.1348	0.4792	0.0010	0.6442	0.9550	0.7839
TE			< .0001	< .0001	0.0034	0.0033	0.0878	0.0178	0.1703
ED			0.0769	0.3997	0.0850	0.0847	0.2861	0.3790	0.2265
PRO × TE			0.0110	0.5339	0.2440	0.2452	0.0006	0.0002	0.0009
PRO × ED			0.7171	0.2174	0.9644	0.9625	0.0830	0.0601	0.0983
TE × ED			0.1987	0.7788	0.2120	0.2128	0.0153	0.0035	0.0404
PRO × TE × ED			0.1256	0.7177	0.1261	0.1262	0.1315	0.0447	0.2434

^a^pH = ruminal pH; DMD = dry matter degradability; SCFA = short-chain fatty acids; ME = the metabolizable energy.

^b^CH_4_:SCFA = methane:short-chain fatty acids ratio; CH_4_:ME = methane:metabolizable energy ratio; CH_4_:OM = methane:organic matter ratio.

^c^SEM = standard error of the mean.

The interaction of both TE and ED at lower protein level (14%) impacted on both CH_4_:SCFA and CH_4_:ME (*p* = 0.0485 and 0.0081) with higher and lower values obtained for nano-chitosan and negative control both at 0.5 ED, respectively. ED did not significantly affect any of the parameters under consideration as used under lower protein levels, however, TE affected pH (*p* =  < 0.0001) and DMD (*p* = 0.0002). At crude protein level of 18%, the interaction TE x ED showed a similar effect on rumen fermentation characteristics and CH_4_ conversion efficiency under consideration while TE influenced all the rumen fermentation characteristics (pH, DMD, SCFA, and ME) and CH_4_ conversion efficiency (CH_4_:SCFA, CH_4_:ME, and CH_4_:OM) under consideration while ED only had a significant effect on SCFA (*p* = 0.004), ME (*p* = 0.004), CH_4_:SCFA (*p* = 0.047), and CH_4_:OM (*p* = 0.041).

## Discussion

The crude protein used was within the limit for ruminant production and the values obtained from the characterization of the nano-encapsulation were < 1 µm, which are within the range for a particle to be referred to as nano. The average size of the nanoparticles as stated by Van et al.^48^ was 89.8 nm and this was higher than that used in this study while the PDI of 0.225 reported was close to that obtained in this study. The size is also close to that of Sathiyabama and Parthasarathy^49^ who had posited that chitosan nanoparticles prepared through ionic gelation using sodium tripolyphosphate yielded nanoparticles had variable size ranges of 250–400 nm. In addition, they reported that biological preparation yields sizes less than 100 nm. This heterogeneity in sizes affects their physico-chemical characters^50^.

Gas production is a pointer to the degree of substrate degradation in the rumen and its increased production is attributed to increased microbial access to the substrate and consequently, increased degradation of the substrate^51^. Asymptotic gas, lag, and gas produced at 48 h were influenced by the interaction between protein, TE, and ED. This further highlights the importance of these three factors in affecting the action of the microbes, time of colonization and adaptation, and enhancement of ruminal fermentation characteristics^52^. The interaction between protein level and TE was significant at only the 6 h of gas production attesting that there was synergy amid both factors in affecting gas production at an initial time.

The TE, ED, and/or the interaction of both could have an immense impact on feed degradation and expressed through gas production kinetics. This is in view of the fact that the TE alone may not be able to effectively increase microbial access to feed for better degradation if not at the appropriate ED. Therefore, the stimulatory effect of the TE and the use of appropriate ED and protein level could result in increased microbial access and degradation of substrate. Although, the interaction between TE and ED was not affected at 14% protein level, but at 18%, the interaction significantly affected c and lag. In this study, using the same ED of 0.5 for nano-extract and crude extract elicited a higher rate of gas production than when 0.25 mL of crude extract was used, and this could be attributed to rapid degradability or formation of acetate and butyrate^53^ caused by the stimulatory effects of the additive at that dosage^52^. Moreover, the additives at higher dose might have created an environment which favoured the adhesion and colonization of the substrate by microbes thereby increasing the rate of gas production^54^. Lower lag for the use of 0.25 mL nano-chitosan compared to the same ED of nano-extract was probably due to the protection by encapsulation using chitosan to reduce ruminal vulnerability to microbial degradation^55^ resulting in the inability of the additive to assist microbes to quickly adapt to the diet and elicit a rapid fermentation process^56^. This implies that it took a longer time before the ED and TE were effective on the feed therefore feed produced with such protein level, TE, and ED may delay feed intake which will negatively affect production except the protected feed will still be useful post- rumen. Feeding yucca extract at an increased level to lactating dairy cows resulted in increased production of gas at 4 h and 24 h^19^.

The protein level altered the asymptotic gas (b) and the time of adhesion and colonization of microbes to the substrate thereby proving its effect on rumen microbe action. The TE influenced all parameters assessed thus demonstrating its effectiveness on microbial population and degradation. The ED affected the initial delay before biogas production began and gas production at 6 h. The similarity in the rate of gas production between crude protein levels of 14 and 18% could be as a result of the minimum crude protein requirement for optimal functioning of the rumen being met. A report of 6–7% crude protein as the minimum requirement for effective rumen function without any negative effect on feed intake was alluded to by Minson^57^. The more gas produced by control could be as a result of the active ingredients present and its effectiveness in degrading feed, done at the effective ED of 1.0 mL/g DM.

The PRO x TE x ED was effective at 24 h of methane production (mL CH_4_ g/DM incubated) with the lower protein level, nano-chitosan extract at a dose of 0.50 in elevating the gas while at the high protein level, nano-chitosan extract and dose of 0.50 elicited a lowered gas production. The nano-chitosan was effective in its mitigation of methane at a higher protein level thereby proving its ability in the inhibition of microbial pathogens in the intestine^28^ as well as methanogenesis^29^.

The PRO x TE affected all parameters except rate of gas constant, while PRO x ED had no influence on the all the parameters. This reveals that protein and TE possessed a profound influence on the CH_4_ gas production while the dose was not enough to alter the effect. The extract type and dose proved to be effective in the suppression, adhesion, and colonization of microbes.

At the lower crude protein level, the higher volume of CH_4_ at 6 h by crude extract at an ED of 0.25 compared to the volume generated by negative control could be attributed to the increased population of microbial communities responsible for CH_4_ generation. The ED could have just been sufficient to stimulate the growth of the CH_4_ producing microbial communities and not suppress it. This explains why the negative control which was without extract with very limited or no antimicrobial ingredients or phytochemicals with the ability to inhibit CH_4_ producing microbial communities resulting in lower CH_4_ production than the inclusion of crude extract at 0.25 (mL/g DM). The role of phytochemicals on CH_4_ production in sheep in vitro had been made by Jack^51^. Lower CH_4_ production has been attributed to a lower protozoa population as a result of the antimicrobial effect of Yucca extract while higher production is attributed to the non-inhibition of methanogens^58^. The increase in methane in the study of Wang et al.^23^ was adduced to the deglycosylation of saponins from YSE that occurred at the 4 h which would have resulted in microbial inactivation of the saponins by the formation of sapogenin which rather stimulated the microbes. Apparently, the 4 h in particular, is best chosen for better evaluation of the gas produced for more reliable fermentation indices resulting from having the peak population of the bacterial community at that time^59^. This assertion would have been the reason for the increase CH_4_ at 6 h. The lower volume of CH_4_ obtained at 24 h from the negative control compared to the use of nano-chitosan at ED of 0.05 was probably due to the unavailability of additives or the inability to alter microbes that would eventually influence the CH_4_ production^58^. The higher CH_4_ generated by nano-chitosan at ED of 0.05 (mL/g DM) simply points to the effectiveness of the encapsulation which limits ruminal microbial interference of the substrate from degradation.

At higher crude protein level, higher CH_4_ production at 6 h elicited with the use of crude extract at 0.25 (mL/g DM) compared to the volume of gas produced by nano-chitosan used at the same extract dose could be attributed to the stimulatory effect of the crude extract at that dosage to CH_4_ producing microbes. The active ingredients could not have been in sufficient quantity to suppress CH_4_ producing microbes and their production. This implies the stimulation of the CH_4_ producing microbes resulting in increased CH_4_ generation beyond the production elicited by protected active ingredients with limited ruminal microbial interference.

Moreso, crude extract accentuated CH_4_ and was more evident when encapsulated at 48 h across both crude protein levels. The slow release from the encapsulation would have been the reason this additive recorded a higher volume through its defaunation property that encouraged methanogens^58^ while nano-chitosan at a higher protein level mitigated CH_4_ through its antimicrobial property of lysing protozoans and methanogens. The anti-protozoan effects of chitosan though noted to be potentially beneficial^60^, could also be deleterious by inhibiting ruminal bacterial community resulting in reduced digestibility of dry matter, and may result in lowered CH_4_. In addition, Belanche et al.^60^ they had also posited that methane, and protozoal activity is lowered through the action of soluble chitosan which modifies ruminal fermentation to promote propionate production.

The nano-chitosan alone did not elicit much gas compared to crude extract and crude extract encapsulated with chitosan except at the lower crude protein level. Therefore, inferring that the crude extract catalyzed the microbes into producing more gas or more or less an activator of ruminal fermentative processes^61^.

The extract dose affected only asymptotic gas, 48 h CH_4_ g/DM, 24 h and 48 h CH_4_ 100 mL/GP with nano-chitosan suppressing CH_4_ at 0.25 mL dose and nano extract elevating it at 1 mL dosage. The interaction effects at 6 h showed that even at the same dosage of 0.25 mL, the crude extract and nano chitosan were not similar in effects, and this may be attributed to the defaunating impacts of the nano chitosan and the selective lysis of Yucca extract. Chitosan is noted to shift ruminal fermentation toward energetically efficient routes, thus improving in vitro energy efficiency^62^. The protein level did not influence all the parameters assessed, while TE affected all parameters under consideration except for 48 h CH_4_ 100 mL/GP, and ED influenced only 6 h CH_4_ 100 mL/GP. This showed that protein does not contribute much to gas production and nano-chitosan generally at 0.25 dose was effective in mitigating CH_4_ production at higher protein level.

It appeared lower protein in the diet was associated with more methane gas production at 24 h after the initiation of incubation. This is in support of the assertion that proteinous substrates do not give off much gas when compared to carbohydrate substrates^63^. More CH_4_ generated by Nano-extract and at the extract doses of 0.25 mL than others could be due to the stimulatory effect of the extract type on the methane-producing communities at that dose.

The PRO × TE × ED was effective in inhibiting the gas (CO) at a lowered protein level with nano-chitosan extract at a dose of 1 mL, while nano-extract at dose of 1 mL with low protein elevated the gas. Reduced CO production could be adduced to the formation of CH_4_ from the continued oxidation of CO_2_^64^ while increased CO production can be attributed to the favourable environment that the interaction caused through the use of CO dehydrogenase enzymes and acetyl-CoA synthetase by acetogenic bacteria^65,66^.

The TE and ED interaction was influenced at 6 and 24 h thereby indicating that at the higher protein level, the crude extract at a lower dose (0.25 mL) elicited a higher CO gas production, for 6 and 24 h, while nano-extract at the same dose occasioned a lower gas of CO. The TE and ED interaction was significant at 6 and 24 h indicated that extract type and extract dose had a synergistic effect only at the sixth and 24 h. From the 4 h, microbes are usually very vigorous, and this might have influenced the interaction between TE and ED at 6 h. The sustainability of this influence is dependent on the TE and ED through their stimulatory or inhibitory effect.

A higher rate of CO production was obtained when 1 mL dosage of nano-extract was incubated using a lower protein level but with lower value observed with 1 mL nano-chitosan inclusion could be attributed to the early access of microbial community and the associated stimulatory effect which increased degradation compared to a protected additive. The lower CO production at 6 h obtained when nano-extract was included could be in connection with the formation of CH_4_ from the continued oxidation of CO, as CO oxidizes when in contact with H_2_O to H_2_ and CO_2_^64^, and these gases are utilized by methanogens for the formation of CH_4_. The increased CO production for the crude extract can be attributed to the use of CO dehydrogenase enzymes and acetyl-CoA synthetase by acetogenic bacteria to catalyze the reduction of CO_2_ to CO through the carbonyl branch of the pathway acetyl-CoA following Wood-Ljungdahl’s metabolic pathway^65,66^. In addition, Ragsdale^67^ reported that the production of CO was positively related to the production of CH_4_ of the crude extract additive, which may indicate that methanogens maintain a synergy with other microorganisms that produce CO.

At higher crude protein level (18%), the crude extract dose of 0.25 elicited higher CO production at both 6 and 24 h showing its positive relationship with CH_4_ production^67^. Moreover, substrate with crude extract presented the higher CH_4_ production showing more H_2_ oxidation and CO_2_ reduction for the generation CH_4_, and during these processes, CO is produced^68^, translating to more formation of CO, which could be the cause for the higher generation exhibited by the additive. The lower CO at 6 h and 24 h obtained for nano-extract and nano-chitosan, respectively proves the positive relationship between CH_4_ and CO^67^ where nano-chitosan lowered CH_4_ at higher crude protein level in the diet and lowered the CO gas at the same crude protein level.

The TE at similar lower doses brought about an increase and decrease in CO gas at a lower time of 6 h due to their accessibility and action to cause effects. It further needed a higher dosage to mitigate the CO gas while the lower dose of the same extract produced a lower gas therefore indicating that the extract had a profound effect on the CO. More CO produced at a crude protein level of 14%, by Nano-chitosan and control (without extract) at 48 h, and at 1.0 mL compared to the other crude protein levels, TE, and ED, could be attributed to reduced oxidation of CO to methane due to its antimicrobial effects on the microbial communities.

The reduced H_2_S produced at 6 h using ED of 0.25 compared to the volume generated by the crude extract at same dose with 18% crude protein diet could be attributed to antimicrobial properties of nano-chitosan. This is in agreement with the finding of Vazquez-Mendoza et al.^55^ reported that the inclusion of nano-chitosan caused an inhibitory effect or an unfavorable environment for the activity of sulphur reducing bacteria. As further alluded to by Vazquez-Mendoza et al.^55^, reduction in the H_2_S gas production at 6 h with the addition of the nano-chitosan in this study can be attributed to an increase in the activity and growth of the rumen microbes, reflected in a lower rate of H_2_S production, and to the antimicrobial effects that they exert on some groups of rumen microbes, including ciliated protozoa and methanogens^69^, microbes that produce and consume H_2_, a gas used by sulphur reducing bacteria for H_2_S production^70^. This is further alluded to by Elghandour et al.^53^ who specifically stated that the low H_2_S generated due to the antimicrobial effects of nano-chitosan stems from the resultant changes in microbial community structure especially, those of hydrogenogens and methanogens. The stimulatory effect or favourable environment initiated by crude extract could be responsible for the increased production of H_2_S^63,71^. An increase in H_2_S could also be adduced to the concentration of sulfur (S) in the feed and its availability^72^, a positive correlation with CO in the current study as that reported by Techtmann et al.^73^ or due to a higher H_2_ availability caused by limited starch digestion^71^ protein, type of extract and extract dose interaction had profound synergistic effect on H_2_S production at both 6 and 48 h with nano-chitosan at all doses mitigating the gas for both levels of proteins. This interaction proved to be effective with the protein, nano-chitosan and dose in curbing the Sulphur reducing bacteria which metabolizes H_2_S by creating an unfavourable environment thereby abating the gas produced. The PRO × TE effect was evident in c (rate of H_2_S production) with nano-chitosan being effective in reducing the rate and in turn the gas was abated thereby proving its mitigating ability while other extract types accentuated the gas at both protein levels. Protein had significant effect on asymptotic gas, rate of H_2_S gas production, and gas production at 6 and 48 h. Signifying that protein might have afforded the microbes a favourable environment to produce the results observed unlike the situation of CO. The TE showed its influence on all the parameters assessed except lag, thereby demonstrating that the extracts played a profound role in altering the microbial structure and activity and by extension fermentation or production of H_2_S. Nano-chitosan mitigated the H_2_S while crude extract and nano-extract increased it at both crude protein levels. The ED (0.25 mL) for both the nano-chitosan and crude extract were effective at 6 h at the higher protein level unlike at the lower protein level proving its effectiveness in abating or elevating H_2_S gas.

The PRO × TE × ED affected only the CH_4_:ME showing that the protein level, type of extract and extract dose had profound effect on the ratio between CH_4_ and ME. The PRO × TE influenced pH and all the CH_4_ conversion efficiency ratios with SCFA, ME and OM. Higher protein level might have occasioned more ammonia and exacerbated the value of the parameters in the presence of the TE. The TE × ED influenced all the CH_4_ conversion efficiency with nano-chitosan at medium dose of 0.50 mL, and crude extract at a lower dose of 0.25 mL, altering values based on their earlier effect on CH_4_.

The pH was reduced by nano-chitosan (dose of 0.50) to 6.22 when compared to negative control of 7.45. This finding is contrary to that reported by several authors^74,75^. However, the present finding is in agreement with that of Belanche et al.^60^ where chitosan inclusion in the diet led to a reduction in pH. However, for optimal microbial stability, pH ranges between 6.0 and 6.8^76^ is ideal. Hence, in the present study, pH after incubation was optimal for microbial function especially with the use of the nano-chitosan. From our findings, the pH is directly proportional to H_2_S at the lower protein level. Whereas Vazquez-Mendoza et al.^55^ had reported that there is a negative correlation between pH and H_2_S production that is attributed to the protonation of aqueous sulfide which inhibits the activity of sulphur reducing bacteria. Moreso, the reduction in pH might be alluded to an increase in the production of SCFA, since the pH decreases with the increasing accumulation of SCFA, Meehan et al.^77^ as seen in this study, in addition to the possibility of increased lactate concentration^60^. The increase in pH observed in this study for nano-extract and crude extract could be explained by the stimulatory effect of the extracts on acetogenesis. This is in agreement with the findings of Belanche et al.^60^ who in their study had posited that saponin/crude extract from Ivy fruit resulted in increased pH which was attributed to elevated acetate and butyrate concentration while propionate was reduced. Moreover, lactate which controls acidity was lowered and this could have contributed to the elevated pH level for crude extract and nano-extract.

The pH of the environment is vital to the antibacterial activity of chitosan. According to Kong et al.^78^, when pH is below the molecules pKa (6.3–6.5), chitosan becomes polycationic, which causes electrostatic interaction between the chitosan and the anionic components of the microorganism’s surface, while on the other hand, hydrophobic and chelating effects are responsible for antibacterial activity of chitosan when the environment is above the pKa. Results obtained in this study is contrary to that of Kirwan et al.^74^ who noted that supplementing beef heifers with chitosan raised the pH.

One of the main reasons for low degradability in feed evaluation is the presence of lignin which protects carbohydrates from attack by rumen microbes^68^ but for nano-chitosan the property of being antimicrobial would have elicited this reduction. The anti-protozoan effects of nano-chitosan although potentially useful^60^, may also prove harmful where it harms the rumen bacteria with a consequent decline in dry matter digestibility/degradability. This reduction in degradability was likely due to the antimicrobial action of chitosan against ruminal microbes (protozoa and fibrolytic bacteria)^60^. A nonreduction and effect was reported by Anele et al.^79^ for feedstuff incubated with yucca crude extract and this was contrary to the observation in this study for crude extract. From our findings as alluded to by Blummel and Becker^80^, degradability is positively correlated to the rate of gas production of which a higher gas production would invariably result in higher dry matter degradability and vice versa. The higher values of DMD observed for other types of extracts (crude extract and nano-extract) could suffice that they occasioned a higher total gas production compared to nano-chitosan extract.

The methane conversion efficiency ratio in this study showed that the negative control (without extract) was more efficient with SCFA and ME at 14% crude protein and this could be attributed to the degradability of the feedstuff. Gas production is directly proportional to feed degradation. The low amount of feed degraded could have also affected the efficiency of degradability.

The TE influenced all the parameters assessed at 18% crude protein content of the substrate. The extra supply of NH_3_ from the degradation of amine groups in chitosan and increased level of protein in the substrate might have occasioned the slight increase in ruminal pH associated with the inclusion of nano-chitosan^74^ when compared with lower protein level (6.87–7.03 vs. 6.22–6.60) which was still lower than negative control and the nano-extract. The increase in pH could be attributed to the shift in fermentation profile to less efficient routes^29^. The low DMD for nano-chitosan was masterminded by its property of being antimicrobial which could have affected cellulolytic bacteria and protozoan activity thereby causing reduced gas production^80^.

Researchers have stated the relevance of SCFA level to connote energy availability that can furnish about 80% of livestock’s daily energy requirement^81,82^. The lower SCFA in nano-chitosan at the higher protein level indicates the effect of the extract type, which, might have affected the rumen microbes compared with that of crude extract. This is in agreement with the findings of Goiri et al.^83^. The crude extract and nano-encapsulated extract were consistently high for SCFA concentration suggesting that the extract types had a stimulatory effect on the microbes to aid feed fermentation and SCFA production. The predominance of SCFA could be ascribed to an increased proportion of volatile fatty acids^84^. The increase in degradability, as observed in the use of crude extract also was reflected in a greater production of SCFA and ME, which is attributed to a greater degradation of carbohydrates^64,85^. The increase in SCFA and ME with the crude extract alludes to a greater activity of the fibrolytic bacteria^86^ and an increase in the production of propionate, while with the use of nano-chitosan where it decreased, could be attributed to the reduction in other SCFA such as acetate^87^.

The methane conversion efficiency ratio in this study showed that the nano-chitosan was more efficient at the higher protein level. This attests to the anti-methanogenic properties of extract^29^. The nano-chitosan at 0.50 mL reduced the production of methane ratios and this mitigation is associated with the SCFA profile as propionate reduces the availability of H_2_ for methane formation^88^, it also correlates with DMD and GP. In line with the findings of this study, Astudillo-Neira et al.^89^ stated that the calculated variations in CH_4_ *per* unit of SCFA, ME, and OM may reflect the effects on DMD and SCFA, which is related to the composition and degradability of feed carbohydrates. The ED had an influence on the SCFA, ME, and CH4 ratios with SCFA and OM with the medium dosage of 0.50 of nano-chitosan proving very effective in lowering their concentrations. The reason for this cannot be far-fetched as the dosage which had a prior effect on the degradability of substrate as a result of hampered microbial activity in turn affected the parameters in question.

Generally, protein had a significant effect on pH and ME with their concentration being increased slightly with the increase in protein diet/substrate. This observation is contrary to previous in vivo studies^90–92^, which reported that pH was not significantly affected by increasing CP level. The type of extract affected all the parameters except CH4:SCFA and CH4:OM proving that nano-chitosan was effective in lowering the parameters in question while negative control and crude extract elevated them.

## Conclusion

From this study, the addition of chitosan had the potential to reduce the gas production at higher protein level by 24.9%. At a higher protein level also nano-chitosan and crude extract of Yucca reduced methane gas per degraded DM at 48 h up 81.40% and 38.4% respectively. For CO, nano-extract (1.00 mL extract/g DM) lowered the gas production by 18.8% at the lower protein level and the mitigation was highly felt at the higher protein level and low dose up to 64.7%. Nano-chistosan caused a reduction in hydrogen sulphide gas and dry matter degradability at both levels of protein of 71.3% at 14% protein, 81.2% at 18% protein, 29.9% at 14% protein level, and 22.5% at 18% protein respectively. The pH level was reduced by the additives at both protein levels. The effect on DMD by nano chitosan was observed in SCFA and ME, being lowered especially at 0.25- and 0.50-mL extract/g DM and at higher protein level. Methane conversion efficiency ratio in this study showed that the nano chitosan type extract was more efficient in lowering CH_4_ gas, and they recorded the lowest value, especially at a higher protein level.

The potential of nano-chitosan in mitigating obnoxious gases has been proven by this study. However, further studies are needed to investigate their effects on other bioactive substances. This step would throw up more information and elucidate their effect on different animal species, the optimal dose levels, and their mode of action, thus facilitating the utilization of chitosan and chitosan-bioactive composites for animal production purposes and environmentally friendly management systems.

## Data Availability

The datasets used and/or analysed during the current study available from the corresponding author on reasonable request.
